# Are Nonalcoholic Fatty Liver Disease and Bone Mineral Density Associated? — A Cross‐Sectional Study Using Liver Biopsy and Dual‐Energy X‐Ray Absorptiometry

**DOI:** 10.1002/jbm4.10714

**Published:** 2023-01-11

**Authors:** Stinus Gadegaard Hansen, Charlotte Wilhelmina Wernberg, Lea Ladegaard Grønkjær, Birgitte Gade Jacobsen, Tina Di Caterino, Aleksander Krag, Claus Bogh Juhl, Mette Munk Lauridsen, Vikram V. Shanbhogue

**Affiliations:** ^1^ Department of Endocrinology, Sydvestjysk Sygehus Syddansk Universitetshospital Esbjerg Denmark; ^2^ Department of Gastroenterology and Hepatology, Sydvestjysk Sygehus Syddansk Universitetshospital Esbjerg Denmark; ^3^ Open Patient data Explorative Network (OPEN) Odense University Hospital Odense Denmark; ^4^ Department of Pathology Odense University Hospital Odense Denmark; ^5^ Center for Liver Research FLASH (Fibrosis Fatty Liver and Steatohepatitis Research Centre) Odense University Hospital Odense Denmark; ^6^ Steno Diabetes Center Odense University Hospital Odense Denmark

**Keywords:** BONE MINERAL DENSITY, LIVER BIOPSY, NAFLD, OSTEOPOROSIS

## Abstract

There is controversy regarding the association between nonalcoholic fatty liver disease (NAFLD) and osteoporosis. Our study aim was to assess bone mineral density (BMD) in patients with biopsy‐proven NAFLD and examine if the severity of NAFLD affects BMD. A total of 147 adult women (*n* = 108) and men (*n* = 39) aged 18–76 years (mean ± standard deviation [SD] age 45.3 ± 12.5) were recruited in this cross‐sectional study and underwent a liver biopsy and dual‐energy X‐ray absorptiometry (DXA). NAFLD activity score (NAS) based on the degree of steatosis, lobular inflammation and hepatocellular ballooning was used to assess NAFLD severity. The majority of subjects, 53%, had steatosis, 25% had nonalcoholic steatohepatitis (NASH) whereas 23% served as control subjects with no evidence of NAFLD. There were no significant differences in the lumbar spine (1.09 ± 0.12, 1.11 ± 0.18, and 1.12 ± 0.15 g/cm^2^, *p* = 0.69, in controls, steatosis, and NASH, respectively) or hip BMD (1.10 ± 0.15, 1.12 ± 0.13, and 1.09 ± 0.13 g/cm^2^, *p* = 0.48, in controls, steatosis, and NASH, respectively) between the groups. Adjusting for age, gender, body mass index, and diabetes in multiple regression models did not alter the results. There was no correlation between NAS and neither lumbar spine BMD (*r* = 0.06, *p* = 0.471), nor hip BMD (*r* = −0.03, *p* = 0.716). In conclusion, BMD was similar across the spectrum of NAFLD in both genders and not related to the severity of the underlying histological lesions, suggesting that neither steatosis nor NASH exerts a detrimental effect on BMD in these relatively young patients. © 2022 The Authors. *JBMR Plus* published by Wiley Periodicals LLC on behalf of American Society for Bone and Mineral Research.

## Introduction

Nonalcoholic fatty liver disease (NAFLD) is the most common liver disorder in Western countries encompassing a spectrum of liver damage ranging from steatosis (increased liver fat without inflammation) and nonalcoholic steatohepatitis (NASH; increased liver fat with inflammation and hepatocellular injury) when no other causes for secondary hepatic fat accumulation are present. Although the pathogenesis of NAFLD has not been fully elucidated, insulin resistance has been implicated as the key mechanism leading to hepatic steatosis with additional oxidative injury and an inflammatory cascade believed to play integral roles in the necroinflammatory component of steatohepatitis.^(^ ^1^ ^)^

Osteoporosis is a common disease characterized by bone loss leading to low bone mass, microarchitectural disruption, and increased skeletal fragility predisposing to an increased risk of fractures. Chronic inflammation has been purported as a risk factor for osteoporosis. Indeed, cytokine activation in chronic inflammatory states such as rheumatoid arthritis, systemic lupus erythematosus, inflammatory bowel disease, primary biliary cirrhosis and chronic viral hepatitis has been associated with low bone mass and osteoporosis.^(^ ^2^ ^)^

In line with this theory, it has been hypothesized that NASH, a chronic inflammatory state, may be associated with low bone mass. In a study of 102 adult patients with NAFLD, Purnak and colleagues^(^ ^3^ ^)^ found that in comparison to healthy control subjects, presence of NASH but not simple steatosis, was associated with low bone mineral density (BMD). Several other studies have found a negative association between steatosis and BMD in men and women,^(^ ^4^ ^)^ postmenopausal women,^(^ ^5^ ^)^ and obese children,^(^ ^6^, ^7^ ^)^ and a negative association between NASH and BMD in obese children.^(^ ^6^ ^)^ However, the evidence till date has been far from convincing, with some studies reporting a higher lumbar BMD in patients with NASH.^(^ ^8^ ^)^ In a recent meta‐analysis including 1276 participants, 638 patients with NAFLD, Upala and colleagues^(^ ^9^ ^)^ found no significant differences in BMD between patients with NAFLD and control subjects.

One of the main limitations fueling the controversy between the association of NAFLD and BMD is that most studies till date have based the diagnosis of NAFLD on ultrasonographic evidence of hepatic steatosis, with an elevated alanine aminotransferase (ALT) serving as a surrogate marker for the presence of NASH. As already alluded to, NAFLD encompasses a spectrum of distinct conditions with variable severity, with liver biopsy remaining the gold standard to distinguish simple steatosis from NASH with inflammation in addition to grading of fibrosis.

Our aim was to assess BMD in patients with biopsy‐proven NAFLD and examine if the severity of NAFLD in obese subjects affects BMD. Based on previous literature we hypothesized NAFLD will be associated with a low BMD.

## Patients and Methods

### Patients

This cross‐sectional study was conducted at University Hospital, Southwest Jutland between August 2018 and December 2021 after ethical approval from the Regional Health Ethics Committee of Southern Denmark (ID S‐20170210). All participants provided written informed consent and the study was performed according to the guidelines from the Declaration of Helsinki. All participants had a body mass index (BMI) ≥35 kg/m^2^ and were accordingly at high risk of NAFLD. None of the participants reported an alcohol consumption above 84 g or 168 g per week for women and men, respectively, or the use of hepatotoxic medications. A questionnaire was used to assess details of medical history including menopausal status in women, diagnosis of diabetes, other obesity related conditions, and medications.

### Liver biopsy and histological analyses

Liver biopsies were obtained during the morning hours on fasting patients using a Menghini suction needle. Histological assessment was performed by a single pathologist (TC) using NAS (NAS‐CRN) for steatosis (0–3), ballooning (0–2), lobular inflammation (0–3), and Kleiner fibrosis grading for fibrosis (0–4).^(^ ^10^ ^)^ Accordingly, NAS range from 0 to 8, and Kleiner fibrosis stage range from 0 to 4, where 4 is liver cirrhosis.

### Anthropometrics

Body weight and height was measured in indoor clothing without shoes to the nearest 0.1 kg using a scale (Tanita) and to the nearest 0.1 cm on a wall‐mounted stadiometer, respectively. A measuring tape was used to measure hip and waist circumferences to the nearest centimeter.

### BMD

Dual‐energy X‐ray absorptiometry (DXA) (Hologic Horizon, Waltham, MA, USA) was used to measure areal BMD (aBMD) at the lumbar spine (L_1_–L_4_) and total hip region. The measurements were obtained no further than a fortnight form the liver biopsy. Lumbar spine BMD was not available in two subjects.

### Blood samples

A venous blood sample was obtained after an overnight fast and analyzed at the Clinical Biochemical Department at UHSD. Ferritin, 25‐hydroxy vitamin D, glycated hemoglobin A1c (HbA1c), insulin, glucose, alanine aminotransferase (ALT), and aspartate aminotransferase (AST) were measured using standardized assays. The homeostatic model assessment for insulin resistance (HOMA‐IR2) was calculated from fasting glucose and insulin using the Oxford HOMA calculator (www.dtu.ox.ac.uk/homacalculator). Ferritin was not available in one and insulin in two subjects.

### Statistical analyses

Statistical analysis was performed using SPSS statistical package version 28.0.1.0 (IBM SPSS Statistics; IBM Corp, Armonk, NewYork, USA). Normality was evaluated using normal probability plots and confirmed objectively using the Shapiro‐Wilk test. All data are expressed as mean ± SD, median (interquartile range [IQR]) or numbers as appropriate. Comparisons between groups were done using chi‐square test for categorical variables and analysis of covariance or independent‐samples median test for normally or non‐normally distributed continuous variables, respectively. Multiple linear regression analyses with lumbar spine BMD or total hip BMD as dependent variables and groups (categorized as 0 control subjects without NAFLD, 1 steatosis, 2 NASH) as independent variable was used to assess differences between the groups adjusted for age, gender, diabetes (0 no diabetes, 1 diabetes) and BMI. Histograms and normal probability plots of residuals were used to check model assumptions. BMI and waist hip ratio were assessed separately in the model to avoid multicollinearity. Bivariate Pearson correlation (for normally distributed data) or Spearman's rank correlation (for non‐normally distributed data) were used to assess the strength and direction of the relationship between lumbar spine/hip BMD, NAS, ferritin, HOMA‐IR, and HbA1c. All tests were two‐tailed and *p* values <0.05 were considered statistically significant.

## Results

A total of 147 adult women (*n* = 108) and men (*n* = 39) aged 18–76 years (mean 45.3 ± 12.5 years) were recruited and underwent a liver biopsy, DXA scan, and the relevant biochemical investigations. Although 23% served as control subjects with no evidence of NAFLD, 53%, had steatosis and 25% had NASH (Table 1). Subjects with steatosis and NASH tended to be older than the control group. There were more women compared to men in all the three groups (*p* = 0.02). Although less than a quarter in the control group and up to two‐thirds of the women in the steatosis and NASH groups were postmenopausal, this was not statistically significant (*p* = 0.11). The prevalence of diabetes, although similar between the steatosis and NASH groups (23% versus 38%), was significantly higher than in the control group where none of the subjects had diabetes (*p* < 0.001). Although subjects in all three groups had similar BMI, subjects with steatosis and NASH had significantly higher waist hip ratio than control subjects (*p* < 0.001).

**Table 1:** General Characteristics of the Study Population, Biochemistry, and Bone Mineral Density

Characteristic	Control	Steatosis	NASH	*p*
Number, *n* (%)	32 (21.7)	78 (53.1)	37 (25.2)	
Age (years), mean ± SD	40.5 ± 11.0	46.8 ± 13.0	46.4 ± 11.8	0.05
Gender (F/M), *n*	28/4	50/28	30/7	0.02
Menopausal status (pre/post), *n*	23/5	30/20	17/11	0.11
Current smoking yes/no, *n*	2/30	14/64	5/32	0.63
BMI (kg/m^2^), mean ± SD	42.9 ± 5.2	42.8 ± 5.7	42.6 ± 5.3	0.96
Waist hip ratio, mean ± SD	0.84 ± 0.09	0.93 ± 0.11	0.91 ± 0.11	<0.001^a^
Diabetes, *n* (%)	0	18 (23.1)	14 (37.8)	<0.001^b^
Hypertension, *n* (%)	9 (28.1)	33 (42.3)	16 (43.2)	0.38
HOMA‐IR2, median (IQR)	2.3 (1.9)	3.2 (2.0)	4.4 (4.7)	<0.001^c^
Vitamin D (nmol/L) (50–160 nmol/L), mean ± SD	63.6 ± 27.6	56.0 ± 25.3	59.8 ± 23.1	0.36
Ferritin (μg/L) (15–180 μg/L), median (IQR)	79 (75)	88 (98)	116 (115)	0.12
HbA1c (mmol/mol) (<48 mmol/mol), median (IQR)	35 (5)	39 (7)	41 (17)	0.004^d^
ALT (U/L) (10–45 U/L), mean ± SD	28 ± 22	41 ± 24	61 ± 27	<0.001 ^e^

Data are expressed as mean ± SD, median (IQR), or numbers as appropriate. HOMA‐IR2 homeostatic model assessment for insulin resistance. ALT = alanine aminotransferase.

^a^ Control versus steatosis (*p* < 0.001) and versus NASH (*p* = 0.005). Steatosis versus NASH (*p* = 0.404).

^b^ Control versus steatosis (*p* < 0.001) and versus NASH (*p* < 0.001). Steatosis versus NASH (*p* = 0.099).

^c^ Control versus steatosis (*p* = 0.003) and versus NASH (*p* < 0.001). Steatosis versus NASH (*p* = 0.017).

^d^ Control versus steatosis (*p* = 0.001) and versus NASH (*p* = 0.006). Steatosis versus NASH (*p* = 0.428).

^e^ Control versus steatosis (*p* = 0.04) and versus NASH (*p* < 0.001). Steatosis versus NASH (*p* < 0.001).

Subjects with steatosis and NASH had significantly higher ALT, HOMA‐IR2, and HbA1c values than the control subjects (*p* < 0.001, *p* < 0.001, and *p* = 0.004, respectively) whereas there was no significant difference in 25‐hydroxy vitamin D or ferritin levels between the three groups.

### BMD

**Fig. 1:**
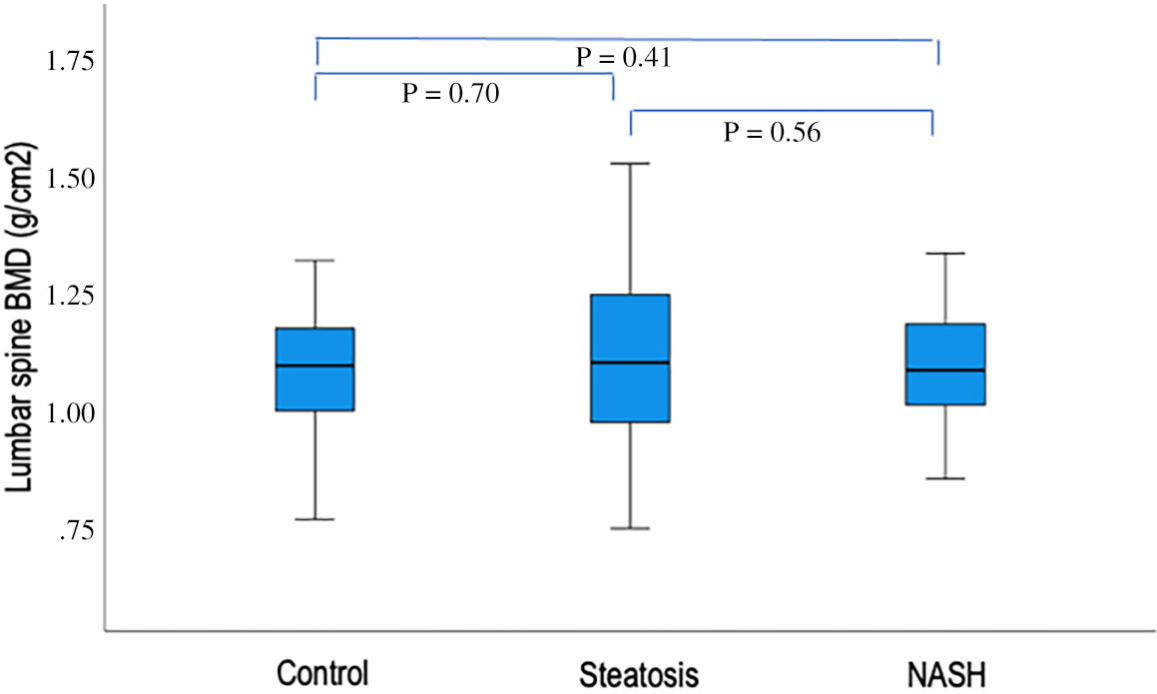
Box‐and‐whisker plots depicting the median, 25th to 75th percentiles, and 5th and 95th percentiles of lumbar spine BMD in the three groups.

**Fig. 2:**
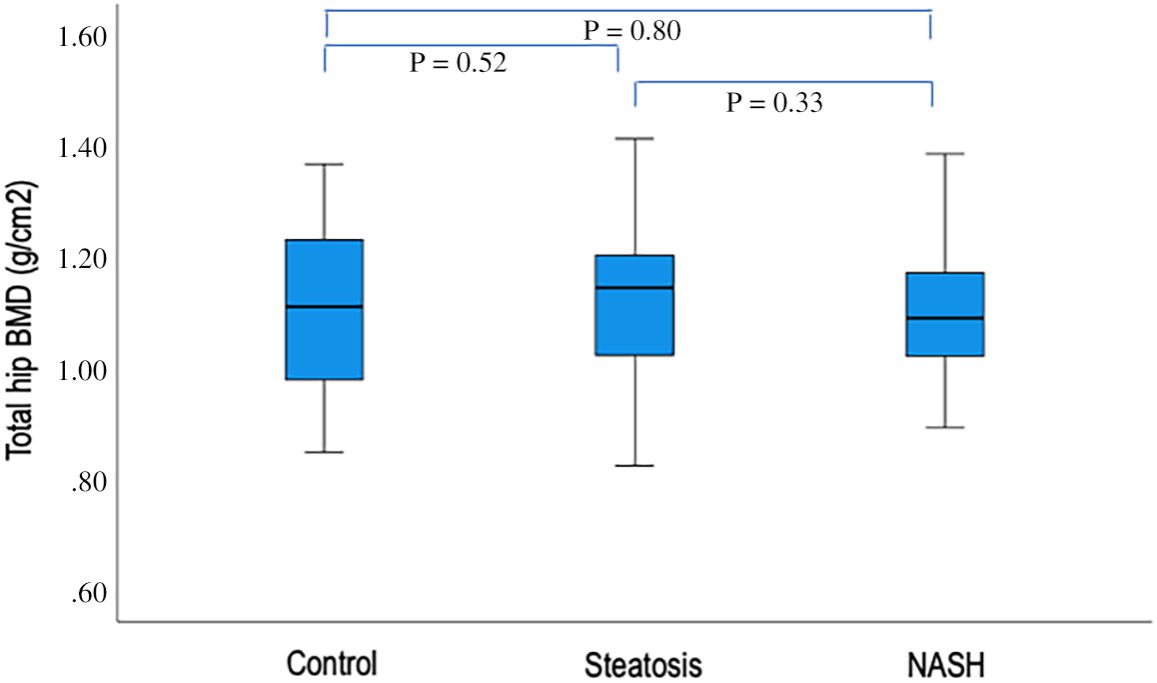
Box‐and‐whisker plots depicting the median, 25th to 75th percentiles, and 5th and 95th percentiles of total hip BMD in the three groups.

There were no significant differences in the lumbar spine (1.09 ± 0.12, 1.11 ± 0.18, and 1.12 ± 0.15 g/cm^2^, *p* = 0.69, in controls, steatosis and NASH, respectively) or total hip BMD (1.10 ± 0.15, 1.12 ± 0.13, and 1.09 ± 0.13 g/cm^2^, *p* = 0.48, in controls, steatosis, and NASH, respectively) measures between the groups (Figs. 1 and 2). Adjusting for age, gender, BMI, and diabetes in multiple regression models did not change the results (Tables 2 and 3).

**Table 2:** Summary of the Regression Analysis of the Effect of the Study Variables on Lumbar Spine BMD

	β coefficient (unstandardized)	β coefficient (standardized	95% CI	*p*
NAFLD^a^	0.002	0.010	−0.039 to 0.043	0.909
Gender^b^	−0.012	−0.031	−0.073 to 0.050	0.713
Age	0.001	0.112	−0.001 to 0.004	0.216
BMI	0.004	0.130s	−0.001 to 0.009	0.142
Diabetes^c^	0.056	0.142	−0.013 to 0.014	0.109

^a^ Categorized as 0 control subjects without NAFLD, 1 steatosis, 2 NASH.

^b^ Categorized as 0 women, 1 men.

^c^ Categorized as 0 no diabetes, 1 diabetes.

**Table 3:** Summary of the Regression Analysis of the Effect of the Study Variables Total Hip BMD

	β coefficient (unstandardized)	β coefficient (standardized	95% CI	*p*
NAFLD^a^	−0.002	−0.112	−0.033 to 0.029	0.911
Gender^b^	0.059	0.195	0.012 to 0.105	0.014
Age	−0.003	−0.271	−0.005 to −0.001	0.001
BMI	0.005	0.208	−0.001 to 0.009	0.142
Diabetes^c^	0.024	0.076	0.001 to 0.009	0.011

^a^ Categorized as 0 control subjects without NAFLD, 1 steatosis, 2 NASH.

^b^ Categorized as 0 women, 1 men.

^c^ Categorized as 0 no diabetes, 1 diabetes.

### Correlations

We found that HOMA‐IR correlated positively with hip BMD (*r* = 0.17, *p* = 0.042), whereas there was a positive trend between HbA1c and lumbar spine BMD (*r* = 0.15, *p* = 0.073). Although there was no correlation between NAS and neither lumbar spine BMD (*r* = 0.06, *p* = 0.471), nor hip BMD (*r* = −0.03, *p* = 0.716), there was a strong positive correlation between NAS and HOMA‐IR (*r* = 0.45, *p* ≤ 0.001) and ALT (*r* = 0.63, *p* ≤ 0.001). There was no correlation between ferritin and lumbar spine BMD (*r* = 0.13, *p* = 0.116), hip BMD (*r* = −0.01, *p* = 0.956), between HbA1c and hip BMD (*r* = 0.08, *p* = 0.309), and between HOMA‐IR and lumbar spine BMD (*r* = 0.06, *p* = 0.491).

## Discussion

To the best of our knowledge, this is the largest study assessing bone mass in adult obese subjects with histologically proven NAFLD. We found no significant difference in BMD, neither at the lumbar spine nor at the hip, in subjects with steatosis and NASH in comparison to obese controls, in spite of accounting for effects of age, gender, diabetes status, and BMI. Further, there was no association between the severity of the histopathological lesions in NAFLD and BMD. In addition, we also found a robust correlation between HOMA‐IR2, a surrogate estimate for insulin resistance, and NAFLD severity scores indicating increasing insulin resistance in subjects with more severe NAFLD.

Our findings are in contrast to the biopsy proven NAFLD study in adult subjects by Kaya and colleagues^(^ ^8^ ^)^ that also assessed the association with BMD. In this small study of 38 patients with histological evidence of NASH and 42 healthy control subjects, the authors reported a higher lumbar spine BMD but not femoral BMD in patients with NASH. They purported that the elevated vitamin D levels seen in patients with NASH in comparison to healthy controls was the main responsible factor for the differences in BMD.^(^ ^8^ ^)^ Although there was no difference in vitamin D between the groups in our study, we had a preponderance of women (almost 64%) as opposed to the aforementioned study^(^ ^8^ ^)^ where 66% of the study cohort were men. Though we neither found any difference in the overall results when adjusting for gender nor in subgroup analysis only in men (data not shown), there was an unequal distribution of men in the various groups in our study with only 10% men in the group without evidence of NAFLD, making it challenging to make reliable comparisons with our study. In a more recent study of 6634 adult subjects where liver ultrasonography was used to detect fatty changes in the lever, Lee and colleagues^(^ ^11^ ^)^ reported a sex‐specific association between hepatic steatosis and BMD, with a negative association in men but a positive association in women. Whether there is a gender predilection and if this is related to the differences between men and women in bone structure and strength, body fat composition or sex hormone levels is uncertain.

Higher BMD as reported by the aforementioned study by Kaya and colleagues^(^ ^8^ ^)^ would suggest that men with NASH have better bone health and theoretically lower fracture risk. However, in a cross‐sectional study of 7797 Chinese adults aged 40 years and above, 2352 of whom had NAFLD diagnosed by hepatic ultrasonography, Li and colleagues^(^ ^12^ ^)^ reported that the presence of steatosis was significantly associated with osteoporotic fractures in men but not women, independently of other potential risk factors such as age, smoking, physical activity, BMI, presence of diabetes, and steroid use. In a recent register‐based retrospective cohort study of 50,689 adult patients with an International Classification of Diseases and Related Health Problems, 10th Revision (ICD‐10) diagnosis of NAFLD, Loosen and colleagues^(^ ^13^ ^)^ reported that during a follow‐up of 10 years, the incidence of osteoporosis as well as osteoporotic and non‐osteoporotic fractures was significantly higher in patients with NAFLD. Although this association was seen in both genders, it was stronger in women compared to men. In a recent population‐based cohort study using national registry, Wester and colleagues^(^ ^14^ ^)^ found a marginally higher incidence of all fractures but not osteoporotic fractures in persons with NAFLD in comparison to the general population. The potential link between NAFLD, osteoporosis and fracture risk remain unclarified, and the available evidence is weakened by limitations related to accurate diagnosis of NAFLD and confounding factors related to gender and comorbidities. In addition, the accuracy of BMD assessment with DXA declines with increasing BMI, which is of importance in our patient population where all of those with NAFLD are overweight or obese.^(^ ^15^ ^)^

We found no significant correlation between the histological severity of hepatic steatosis, necroinflammation and fibrosis and lumbar spine or hip BMD. Although the scarcity of studies assessing BMD and histologically diagnosed NAFLD limits comparisons, evidence from other studies using liver ultrasonography present conflicting results. Lee and colleagues^(^ ^11^ ^)^ reported that mild steatosis had no effect on BMD, but more advanced steatosis had a detrimental impact on femoral neck BMD in men but a positive effect on lumbar BMD in postmenopausal women. Purnak and colleagues^(^ ^3^ ^)^ found no significant relationship between the degree of hepatic steatosis and BMD, but lower BMD in a subgroup of patients assumed to have NASH based on the presence of elevated ALT and high‐sensitivity C‐reactive protein. Liver biopsy remains the gold standard in NAFLD patients for grading the severity of steatohepatitis based on the increasing degree of steatosis, hepatocyte ballooning, and degree of inflammatory foci. However, the inflammatory component extends across the spectrum of steatosis and NASH, limiting the utility of systemic inflammatory markers in predicting the severity of NAFLD.

We found higher levels of insulin resistance with increasing severity of NAFLD. The pathophysiology of NAFLD is multifactorial and not fully elucidated but the most widely supported theory implicates insulin resistance and cytokine activation leading to a chronic inflammatory state as key mechanisms leading to NASH.^(^ ^16^ ^)^ There is some controversy regarding the effect of insulin on bone. Although some in vitro studies indicate that insulin stimulates bone formation^(^ ^17^ ^)^ and bone resorption,^(^ ^18^ ^)^ clinical studies across a wide range of hyperinsulinemic states such as polycystic ovarian syndrome, impaired glucose tolerance, and type 2 diabetes feature the consistent finding of high BMD.^(^ ^19^, ^20^, ^21^ ^)^ Thus, the higher prevalence of type 2 diabetes in patients with NAFLD in comparison to control subjects and tendency toward higher prevalence in patients with NASH than in those with steatosis in our study could have potentially impacted/mitigated differences in BMD between the groups. On the other hand, the important role of dysregulated inflammatory signaling pathways in the development and progression of NAFLD had been demonstrated, potentially linking NAFLD to osteoporosis.^(^ ^22^ ^)^ Chronic inflammation leads to increased bone resorption and suppressed bone formation and is increasingly recognized as a risk factor for osteoporosis.^(^ ^23^ ^)^ Thus, it can be speculated that detrimental effects on the skeleton due to the chronic inflammatory state are counteracted to a large extent, by the hyperinsulinemia secondary to insulin resistance.

Although we used histology to assess the severity of NAFLD in a fairly large cohort of women and men, certain limitations need to be addressed. First, the cross‐sectional nature of the study does not allow for addressing the casualty of findings. Second, unequal numbers in the groups and between the genders precludes any firm conclusions on the specific effects in each sex. Third, we used DXA to assess BMD and as alluded to above, this may limit the assessment of bone mass in those with obesity compared to measurement using three‐dimensional techniques such as computed tomography.^(^ ^15^ ^)^ The mean BMI of participants in our study was 42 kg/m^2^ and although there was no difference between BMI across the three groups this may have affected measurement of BMD and the ability to explore the association with NAFLD.

In conclusion, BMD was similar across the spectrum of NAFLD in both genders. Although there was no correlation between the severity of the underlying histological lesions and bone mass, subjects with more severe liver disease were increasingly insulin resistant and that could partly account for preserved BMD. In conjunction with recent epidemiologic data suggesting no increased risk of osteoporotic fractures in patients with NAFLD in comparison to the background population,^(^ ^14^ ^)^ our findings suggest that screening for osteoporosis in these relatively young patients may not be warranted.

### Peer Review

The peer review history for this article is available at https://publons.com/publon/10.1002/jbm4.10714.
